# Endometrial thickness is an independent risk factor of hypertensive disorders of pregnancy: a retrospective study of 13,458 patients in frozen-thawed embryo transfers

**DOI:** 10.1186/s12958-022-00965-8

**Published:** 2022-06-28

**Authors:** Meng Zhang, Jing Li, Xiao Fu, Yiting Zhang, Tao Zhang, Bingjie Wu, Xinyue Han, Shanshan Gao

**Affiliations:** 1 Center for Reproductive Medicine, The Second Hospital, Cheeloo College of Medicine, Shandong University, Jinan, 250012 Shandong China; 2 Key laboratory of Reproductive Endocrinology of Ministry of Education, Shandong University, Jinan, 250012 Shandong China; 3 Shandong Key Laboratory of Reproductive Medicine, Jinan, 250012 Shandong China; 4 Shandong Provincial Clinical Research Center for Reproductive Health, Jinan, 250012 Shandong China; 5 National Research Center for Assisted Reproductive Technology and Reproductive Genetics, Shandong University, Jinan, 250012 Shandong China; 6 Department of Biostatistics, School of Public Health, Cheeloo College of Medicine, Shandong University, Jinan, China

**Keywords:** IVF/ICSI, FET, Ultrasound measurements of endometrium thickness, Natural cycles, Hormone programmed cycles, Obstetric complications

## Abstract

**Background:**

Hypertensive disorders of pregnancy (HDP) are an important cause of maternal and fetal mortality, and its potential risk factors are still being explored. Endometrial thickness (EMT), as one of the important monitoring indicators of endometrial receptivity, has been confirmed to be related to the incidence of HDP in fresh embryo transfer. Our study was designed to investigate whether endometrial thickness is associated with the risk of hypertensive disorders of pregnancy in frozen-thawed embryo transfer (FET).

**Methods:**

This respective cohort study enrolled 13,458 women who received vitrified embryo transfer and had a singleton delivery in the Reproductive Hospital affiliated to Shandong University from January 2015 to December 2019. We set strict screening criteria and obtained the information from the hospital electronic medical system. Statistical methods including logistic regression analysis, receiver operating characteristic curve and restricted cubic spline were used to evaluate the relationship between endometrial thickness and the incidence of pregnancy-induced hypertension.

**Results:**

The incidences of HDP in a thin endometrial thickness group (< 0.8 cm) and a thick endometrial thickness group (> 1.2 cm) were significantly greater than in a reference group (0.8 cm–1.2 cm) (7.98 and 5.24% vs 4.59%, *P* <  0.001). A nonlinear relationship between endometrial thickness and risk of hypertensive disorders of pregnancy was examined by restricted cubic spline (P <  0.001). The thin endometrial thickness and thick endometrial thickness groups were significantly associated with the risk of HDP after adjusting for confounding variables by stepwise logistic regression analysis. Subsequently, subgroup logistic regression analysis based on endometrial preparation regimens showed that thin endometria were still significantly associated with a higher morbidity rate in the artificial cycle group, while in the natural cycle group, thick endometria were closely associated with increased morbidity.

**Conclusion:**

Our study manifested that both the thin and thick endometria were associated with an increased risk of hypertensive disorders of pregnancy in frozen embryo transfer cycles. Reproductive clinicians should focus on adjusting endometrial thickness in different preparation regimens; and obstetricians should be mindful of the risk of hypertension during pregnancy, when women with thin (< 0.8 cm) or excessively thicker (> 1.2 cm) endometrial thickness achieve pregnancy through frozen-thawed embryo transfer.

## Background

With refinements in assisted reproductive technology (ART), births following in vitro fertilization (IVF) have increased dramatically over the last two decades [1, 2]. Therefore, a higher pregnancy rate is no longer the focus of research; attention has turned to better obstetric and perinatal outcomes [3]. However, compared with spontaneous conception, pregnancies conceived by ART are associated with increased risk of obstetric complications and lower birth weight [4]. Now, several candidate risk factors including multiple pregnancy, hormonal stimulation, embryo culture and biopsy, and fresh/frozen embryo transfer (ET) policy have been revealed [5–7].

Frozen embryo transfer (FET) could increase the accumulated chance of pregnancy for patients with surplus embryos, and facilitate delayed transfer in cases of premature progesterone rise, ovarian hyper-stimulation syndrome, or preimplantation genetic screening after blastocyst biopsy [8–10]. Although a higher clinical pregnancy rate, decreased risks of small for gestational age, low birth weight, and preterm delivery in FET cycles have been reported over those of fresh ET, evidence from two recent meta-analyses shows some adverse obstetrics and perinatal outcomes after FET including pregnancy-induced hypertension, large for gestational age (LGA), and postpartum hemorrhage [3, 11]. These results indicate that FET and fresh ET have their respective advantages and disadvantages in obstetric and perinatal outcomes. Further studies have investigated the possible risk factors within fresh ET or FET cycles including high estrogen level, absence of a corpus luteum (CL), and thin endometrial thickness [12–15]. However, reasons for the impact of FET procedures on obstetrics and perinatal outcome require further study.

EMT is a key factor influencing endometrial receptivity and is a predictor of early pregnancy outcomes in ET cycles [16]. Furthermore, our previous studies demonstrate that thin EMT is associated with small for gestational age infants and hypertensive disorders of pregnancy in fresh ET cycles [17, 18]. Another study from our clinic shows that women with a singleton delivery after FET in artificial cycles had an elevated risk of pre-eclampsia and postpartum hemorrhage compared with those in natural cycles [19]. Therefore, the associations between EMT and obstetrics/perinatal outcomes after FET with different endometrial preparation methods require urgent analysis.

The aim of this study was to investigate the association between EMT and the incidence of hypertensive disorders in singleton deliveries following the transfer of vitrified embryos with programmed or natural endometrial preparation cycles in IVF/ICSI (in vitro fertilization/intracytoplasmic sperm injection).

## Methods

### Study design and patients

This was a retrospective cohort study undertaken in the Reproductive Hospital of Shandong University, to investigate the effect of EMT on the risk of hypertensive diseases of pregnancy (HDP). The study was approved by the Ethics Committee of Reproductive Medicine of Shandong University. Inclusion criteria: (1) patients who were ≤ 40 y and ≥ 20 y (both oocyte retrieval age and embryo transfer age); (2) delivered a singleton infant after 28 weeks gestation; (3) systolic blood pressure < 140 mmHg; diastolic blood pressure < 90 mmHg before FET. Exclusion criteria: (1) preimplantation genetic testing; (2) pregnancies using donor sperm or donor oocyte; (3) chronic hypertension diseases or diabetes mellitus; (4) multiple birth; (5) fetal reduction; (6) vanishing twin syndrome after 14 weeks of gestation [20]; (7) uterine malformations. All basic data were collected from the hospital electronic medical system. Almost all the patients had hysteroscopic examination before their embryo transfer cycles. Women with moderate to severe intrauterine adhesion would accept hysteroscopic surgery to improve their intrauterine environment. Fifty-eight patients were lost to follow-up and two patients lacked vital information and were excluded. Finally, 13,458 patients were selected for the study.

### Natural cycles/hormone programmed cycles

For natural cycles, follicles were monitored by transvaginal ultrasonography from day 8 to 10 day of the menstrual cycle. Serum or urine luteinizing hormone, and serum progesterone and estrogen were also tested to evaluate the exact ovulation time. Ovulation occurred either spontaneously or was induced by human chorionic gonadotropin. After ovulation was confirmed, the optimal recovery and transfer time of the vitrified embryos was determined according to frozen embryo stage.

For hormone programmed cycles, patients began taking 4–8 mg of oral estradiol for at least 10 days starting from day 2–5 of the menstrual cycle. If EMT was less than0.8 cm, the estradiol dose could be increased and/or the duration of administration could be extended, until EMT reached 0.8 cm, or was < 0.8 cm while the clinicians and the patients recognized it as a relatively maximum thickness; or the clinicians and the patients decided to terminate the current cycle. Then, progesterone would be given to start endometrial transformation.

### Endometrium thickness assessment

For natural cycles, assessment of endometrial thickness was performed on the day that we could decide the (theoretical) ovulation day and the transfer day. While in programming cycles, assessment would take place before starting progesterone support. The maximal distance from one interface of the endometrium-myometrium to the other in the midsagittal plane of the uterus including the cervical canal was measured by ultrasound (GE medical systems, Co., Ltd). For each patient, the endometrium measurement was routinely carried by two ultrasound technicians, one was operator, the other was recorder and checker. And the same ultrasound images were real-time shared by the two technicians during the measurement to prevent operation or measure mistakes. Lastly, the measurements were recorded in the electronic medical system.

### Patients’ follow-up

As mentioned in a previous study [18], the first follow-up was performed around 14 d after FET, and biochemical pregnancy was assessed by measuring the serum HCG-beta subunit. The second follow-up was performed at 5 or 6 weeks after embryo transfer; a clinical pregnancy was confirmed by the presence of a gestational sac by transvaginal ultrasound. An ongoing pregnancy was confirmed at the third follow-up, which was performed at 12th week of gestation (9–10 weeks after ET). Subsequently, the patients would receive telephone surveys and standardized questionnaires by trained nurses. Information would be collected including perinatal complications, gestational weeks, birth date, delivery mode, newborn gender and birth weight, neonatal diseases, treatment, and prognosis. All follow-up information was recorded in the electronic medical records.

The target outcome was HDP, a disease that pregnancy coexists with elevated blood pressure, including gestational hypertension, pre-eclampsia, eclampsia, chronic hypertension complicating pre-eclampsia, as well as chronic hypertension complicating pregnancy. Our study covered only the first three categories. Gestational hypertension is diagnosed as: blood pressure elevated ≥140/90 mmHg after two or more measurements at least 4 h apart in pregnant women with previously normal blood pressure. Pre-eclampsia suggests that newly-systemic complications emerged coexistent with gestational hypertension, including proteinuria (protein ≥300 mg/24 h or a protein-to-creatinine ratio ≥ 0.30), blurred vision, and liver and kidney dysfunction. Moreover, eclampsia is both the onset of convulsions in a pregnant woman with pre-eclampsia without other causative conditions, and also a severe manifestation for HDP [21].

### Statistical analysis

Initially, we performed descriptive statistics and univariate analyses with variables that may influence patient perinatal outcomes according to clinical experience and up-to-date literature. Continuous variables were presented as mean ± standard deviation with one-way analysis of variance (ANOVA) and the between-groups differences were analyzed by LSD post-hoc multiple comparison. Categorical variables were expressed as frequencies and percentages, and the distribution among groups was analyzed by chi-square test or Fisher’s exact test. Secondly, univariate and multivariate regression analyses were used to identify factors associated with the incidence of HDP. Odds ratios (ORs) and 95% confidence intervals (CIs) were calculated to show the relationship between EMT and the risk of HDP after adjusting variables in multivariate regression analyses. All variates in multivariate regression analyses, including maternal age, body mass index (BMI), type of infertility, polycystic ovary syndrome (PCOS), history of intrauterine adhesion, previous birth, number of abortions, EMT, mean arterial pressure (MAP), development of embryos, gestational diabetes mellitus (GDM), cesarean section experience, and the number of embryos. PCOS was defined as a syndrome of ovarian dysfunction with clinical manifestations including menstrual irregularities, signs of androgen excess, and obesity according to the Rotterdam Consensus [22, 23]. Systolic blood pressure, diastolic blood pressure, and BMI, as general parameters, were measured before FET to estimate each patient’s physical condition. Mean arterial pressure was an important index for hemodynamics [24]; it was equal to one-third of the sum of systolic blood pressure plus two-fold diastolic blood pressure in clinical work [25]. GDM could be diagnosis with a 75 g oral glucose tolerance test (OGTT) with one or more existing characteristics including fasting plasma glucose ≥5.1 mmol/L, a 1 h plasma glucose value ≥10.0 mmol/L, and a 2 h plasma glucose value ≥8.5 mmol/L [26]. *P*-values of < 0.05 were considered statistically significant. Discrimination performance was illustrated by a receiver operating characteristic (ROC curve) using the area under the curve (AUC). Restricted cubic spline was used to visualize the relation of predicted EMT with HDP incidence, and an EMT = 0.8 cm was set as the reference value. All statistical analyses were performed by using R v4.1.2.

## Results

A total of 13,458 patients were enrolled for this research, consisting of 827 patients with an EMT <  0.8 cm, 1069 patients with an EMT > 1.2 cm, and 11,562 patients with an EMT between 0.8 cm and 1.2 cm. The group with the EMT of 0.8–1.2 cm was set as the reference group. A total of 8456 women delivered a singleton birth after FET with a natural cycle and 5002 women with an artificial protocol.

All basic characteristics in the three groups classified by EMT are shown in Table 1. There were significant differences were found in birth weight (3.38 ± 0.56 kg vs 3.46 ± 0.51 kg vs 3.45 ± 0.50 kg, *P* <  0.001), cesarean section rate (74.61% vs 69.15% vs 64.36%, P <  0.001), and HDP rate (7.89% vs 4.59% vs 5.24%, P <  0.001) among the three groups according to EMT. Mean EMT of the three groups were 0.72 ± 0.05 cm, 0.97 ± 0.12 cm, and 1.32 ± 0.08 cm, respectively.

**Table 1:** Baseline demographic and clinical characteristics of patients

Variables	Normal EMT	Thin EMT	Thick EMT	*P-*values			
				All	Normal vs thin	Normal vs Thick	Thin vs Thick
**Number**	11,562	827	1069				
**Maternal age (y)**	30.41 (3.97)	31.05 (4.11)	30.51 (3.94)	< 0.001	< 0.001	0.473	0.003
**Maternal BMI (kg/m**^**2**^**)**	23.20 (3.51)	22.87 (3.34)	23.25 (3.42)	0.027	0.010	0.611	0.018
**Paternal age (y)**	31.12 (4.59)	31.76 (5.05)	30.87 (4.49)	< 0.001	< 0.001	0.096	< 0.001
**Paternal BMI (kg/m**^**2**^**)**	25.81 (4.08)	25.88 (4.19)	26.12 (4.14)	0.058	0.618	0.018	0.214
**Type of infertility, n (%)**							
Primary	6365 (55.05)	302 (36.52)	665 (62.21)				
Secondary	5197 (44.95)	525 (63.48)	404 (37.79)	< 0.001	< 0.001	< 0.001	< 0.001
**PCOS, n (%)**	2034 (17.59)	179 (21.64)	97 (9.07)	< 0.001	< 0.001	< 0.001	< 0.001
**Intrauterine adhesion, n (%)**	214 (1.85)	85 (10.28)	4 (0.37)	< 0.001	< 0.001	0.002	< 0.001
**Childbirth experience, n (%)**							
Nullipara	8127 (70.29)	551 (66.63)	726 (67.91)				
Pluripara	3435 (29.71)	276 (33.37)	343 (32.09)	0.029	0.087	0.336	0.999
**Number of abortions, n (%)**							
0	7545 (65.26)	374 (45.22)	749 (70.07)				
1	2825 (24.43)	263 (31.80)	220 (20.58)				
≥ 2	1192 (10.31)	190 (22.97)	100 (9.35)	< 0.001	< 0.001	0.017	< 0.001
**Development of embryo, n (%)**							
Blastocyst	11,367 (98.31)	820 (99.15)	1049 (98.13)				
Cleavage-stage	195 (1.69)	7 (0.85)	20 (1.87)	0.157	0.267	0.999	0.284
**Mean EMT (cm)**	0.97 (0.12)	0.72 (0.05)	1.32 (0.08)	< 0.001	< 0.001	< 0.001	< 0.001
**Delivery model, n (%)**							
Spontaneous labor	3567 (30.85)	210 (25.39)	381 (35.64)				
Cesarean labor	7995 (69.15)	617 (74.61)	688 (64.36)	< 0.001	0.003	0.004	< 0.001
**Mean neonatal weight (kg)**	3.46 (0.51)	3.38 (0.56)	3.45 (0.5)	< 0.001	< 0.001	0.587	0.005
**Outcome, n (%)**							
Full-term birth	10,825 (93.63)	758 (91.66)	1007 (94.20)				
Pre-term birth	704 (6.09)	66 (7.98)	59 (5.52)				
Post-term birth	33 (0.29)	3 (0.36)	3 (0.28)	0.215	0.256	0.999	0.285
**HDP, n (%)**	531 (4.59)	66 (7.98)	56 (5.24)	< 0.001	< 0.001	0.999	0.061
**Number of embryos, n (%)**							
1	10,652 (92.13)	774 (93.59)	1000 (93.55)				
> 1	910 (7.87)	53 (6.41)	69 (6.45)	0.093	0.454	0.343	0.999
**History of cesarean delivery, n (%)**	1724 (14.91)	127 (15.36)	155 (14.50)	0.873	0.999	0.999	0.999
**Endometrial preparation regimen, n (%)**							
Natural	7190 (62.19)	350 (42.32)	916 (85.69)				
Artificial	4372 (37.81)	477 (57.68)	153 (14.31)	< 0.001	< 0.001	< 0.001	< 0.001
**GDM, n (%)**	836 (7.23)	71 (8.59)	84 (7.86)	0.288	0.507	0.999	0.999
**Systolic blood pressure (mmHg)**	116.54 (11.89)	115.46 (11.54)	117.09 (11.60)	0.011	0.012	0.146	0.003
**Diastolic blood pressure (mmHg)**	69.66 (8.68)	68.94 (8.41)	70.12 (8.87)	0.013	0.020	0.102	0.003

Values are presented as mean ± standard deviation or n (%)

The between-groups differences in continuous variables are analyzed by LSD post-hoc multiple comparison

*BMI* Body mass index, *EMT* Endometrial thickness, *PCOS* Polycystic ovary syndrome, *HDP* Hypertensive disorders of pregnancy, *GDM* Gestational diabetes mellitus

Table 2 shows the univariate logistic regression analysis regarding related factors possibly associated with the risk of HDP. After adjusting the confounding factors, multivariate logistic regression still suggested that EMT was an independent risk factor of HDP. The increased risk of HDP after FET was associated with an EMT < 0.8 cm (aOR = 1.73; 95% CI, 1.31–2.27, *P* <  0.001) or > 1.2 cm (aOR = 1.39; 95% CI, 1.03–1.85, *P* = 0.028). Our study showed that maternal age (OR = 1.11; 95% CI, 1.02–1.21, *P* = 0.022), BMI (OR = 1.29; 95% CI, 1.20–1.39, *P* <  0.001), and GDM (OR = 1.79; 95% CI, 1.40–2.27, *P* <  0.001) were associated with a higher incidence of HDP after stepwise logistic regression analysis, whereas previous childbirth experience (OR = 0.55; 95% CI, 0.45–0.68, *P* <  0.001) was considered as a protective factor for HDP. For different endometrial preparations, the artificial cycle was associated with a higher incidence of HDP (aOR = 2.13; 95% CI, 1.79–2.52, *P* <  0.001).

**Table 2:** Logistic regression analysis of predictor variables for hypertensive disorders of pregnancy

Variable	Univariate logistic regression		Multivariate logistic regression		Stepwise logistic regression	
	OR (95% CI)	*P*-value	OR (95% CI)	*P*-value	OR (95% CI)	*P*-value
Maternal age	1.02 (0.94–1.10)	0.602	1.10 (1.01–1.20)	0.035	1.11 (1.02–1.21)	0.022
Maternal BMI	1.53 (1.42–1.64)	< 0.001	1.30 (1.21–1.40)	< 0.001	1.29 (1.20–1.39)	< 0.001
MAP	1.78 (1.64–1.94)	< 0.001	1.69 (1.55–1.84)	< 0.001	1.68 (1.54–1.83)	< 0.001
GDM	2.17 (1.71–2.72)	< 0.001	1.82 (1.42–2.30)	< 0.001	1.79 (1.40–2.27)	< 0.001
Type of infertility	0.94 (0.80–1.10)	0.457	1.12 (0.90–1.39)	0.303		
PCOS	1.77 (1.47–2.11)	< 0.001	0.91 (0.74–1.12)	0.378		
Intrauterine adhesion	1.40 (0.85–2.16)	0.154	1.09 (0.65–1.72)	0.733		
Childbirth experience	0.61 (0.51–0.74)	< 0.001	0.57 (0.42–0.75)	< 0.001	0.55 (0.45–0.68)	< 0.001
Number of abortions						
0	Ref					
1	1.12 (0.93–1.34)	0.214	1.00 (0.80–1.24)	0.964		
≥ 2	0.95 (0.72–1.23)	0.711	0.80 (0.58–1.09)	0.156		
Development of embryos	0.63 (0.27–1.25)	0.238	0.91 (0.37–1.93)	0.825		
Endometrial preparation	2.51 (2.14–2.95)	< 0.001	2.19 (1.83–2.64)	< 0.001	2.13 (1.79–2.52)	< 0.001
EMT						
0.8 cm ≤ EMT ≤ 1.2 cm	Ref					
EMT < 0.8 cm	1.80 (1.37–2.33)	< 0.001	1.73 (1.29–2.27)	< 0.001	1.73 (1.31–2.27)	< 0.001
EMT > 1.2 cm	1.15 (0.86–1.51)	0.337	1.40 (1.03–1.86)	0.025	1.39 (1.03–1.85)	0.028
Number of embryos	0.82 (0.59–1.11)	0.224	0.77 (0.54–1.07)	0.138	0.76 (0.54–1.04)	0.095
History of CS	0.67 (0.52–0.86)	0.002	0.90 (0.63–1.27)	0.538		

*BMI* Body mass index, *MAP* Mean arterial pressure, *GDM* Gestational diabetes mellitus, *EMT* Endometrial thickness, *PCOS* Polycystic ovary syndrome, *CS* cesarean delivery

**Fig. 1:**
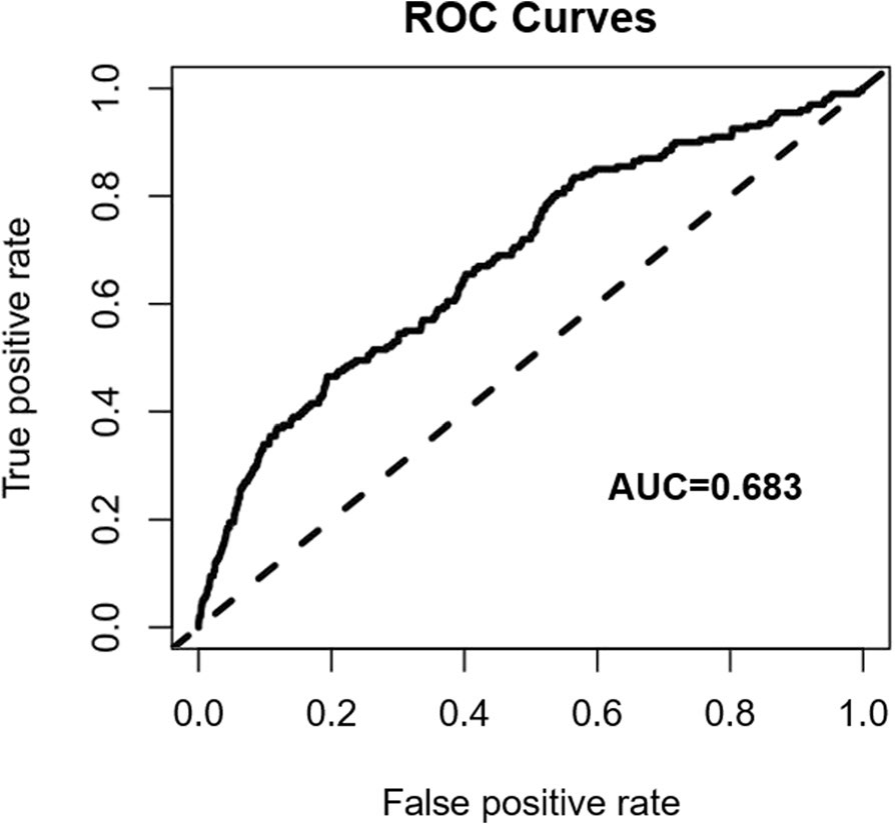
The ROC curve for multivariate stepwise regression analysis model

To evaluate the applicability of the current regression model, a ROC curve was created (Fig. 1). The area AUC reached 0.683, indicating that this model predicted the occurrence of HDP to a certain extent.

**Fig. 2:**
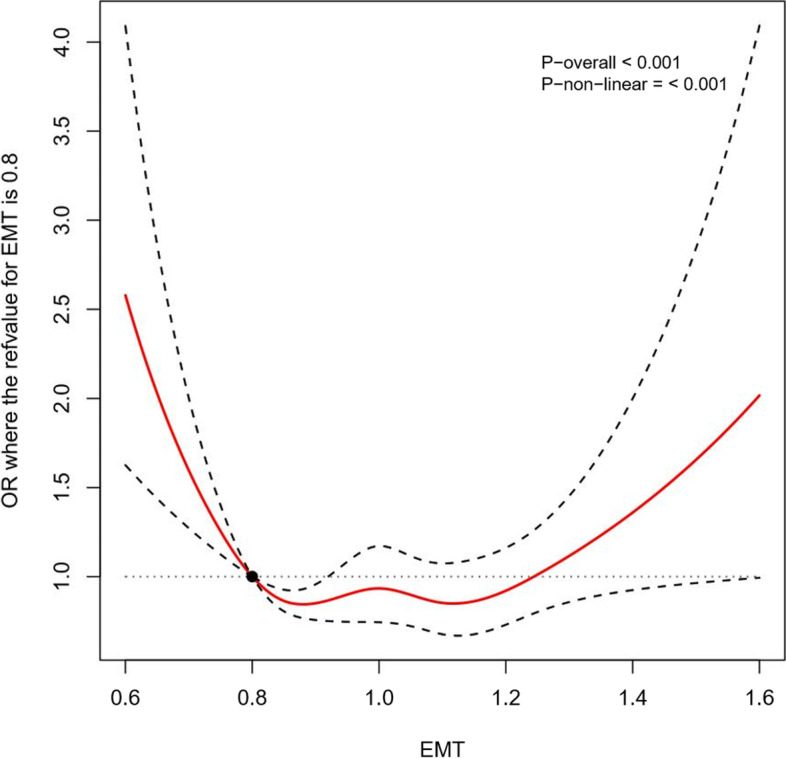
The restricted cubic splines for EMT in association with HDP rate incorporating multivariate stepwise regression analysis model. (EMT = 0.8 cm as reference, knots = 5)

The results of restricted cubic spline (Fig. 2) demonstrated the non-linear relationship between EMT and the HDP rates (*P* <  0.001). With a reduction of EMT, the HDP risk significantly increased. The results showed that patients with an EMT ≤0.6 cm or an EMT around 0.7 cm (0.6 cm < EMT <  0.8 cm) were associated with an increased risk of HDP with ORs of 2.58 (95% CI, 1.63–4.09) and of 2.03 (95% CI, 1.44–2.86), respectively, compared with women with an EMT of 0.8 cm as a reference (Table 4). However, the dose relationship became insignificant with thicker EMT (1.3 cm, 1.4 cm, 1.5 cm, ≥1.6 cm) according to restricted cubic splines (Table 4). The probability of HDP in patients with an EMT of 0.8–1.2 cm reached the low ebb of the curve.

**Fig. 3:**
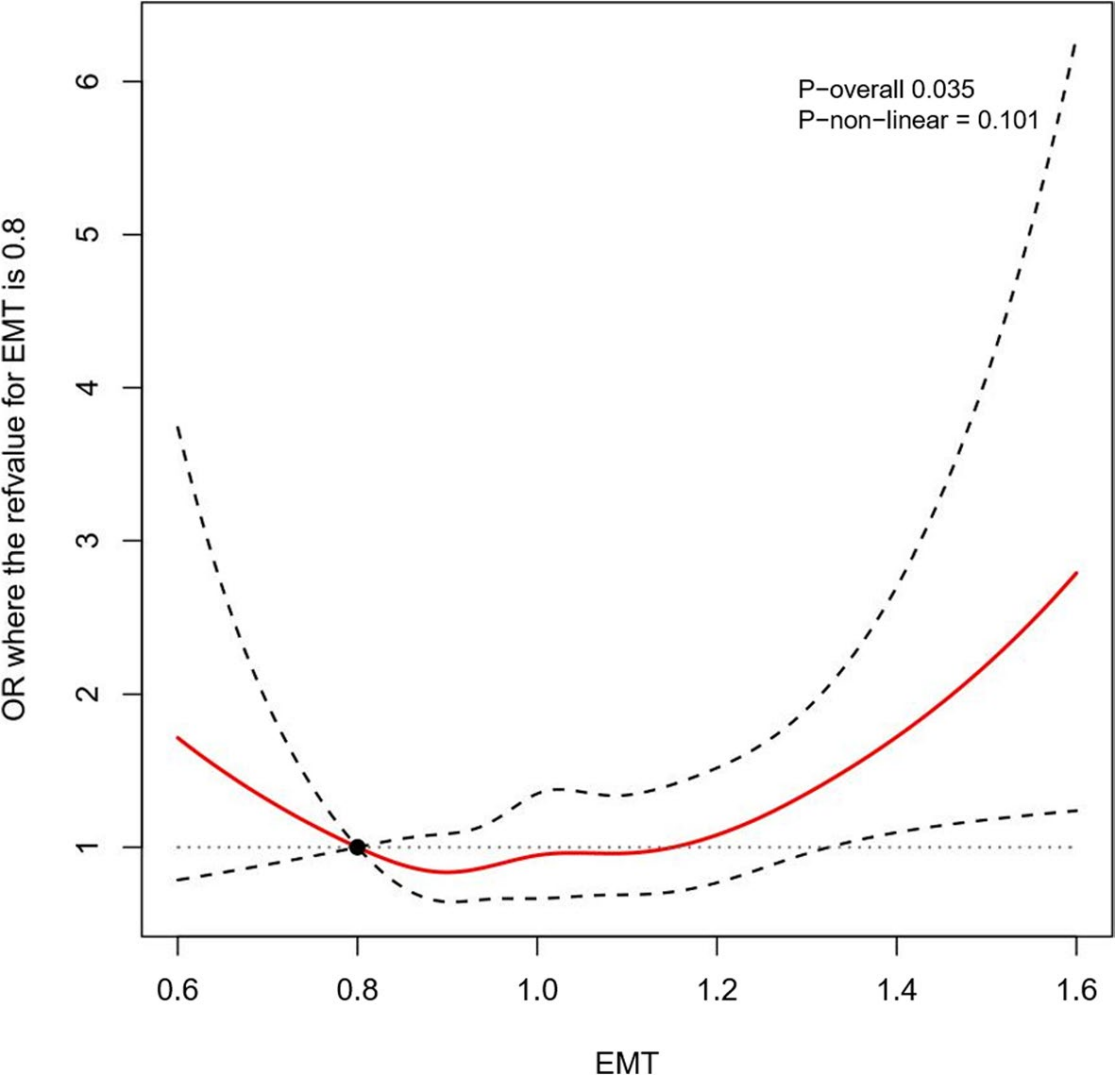
The restricted cubic splines for EMT in association with HDP rate in natural cycle incorporating multivariate stepwise regression analysis model. (EMT = 0.8 cm as reference, knots = 5)

**Fig. 4:**
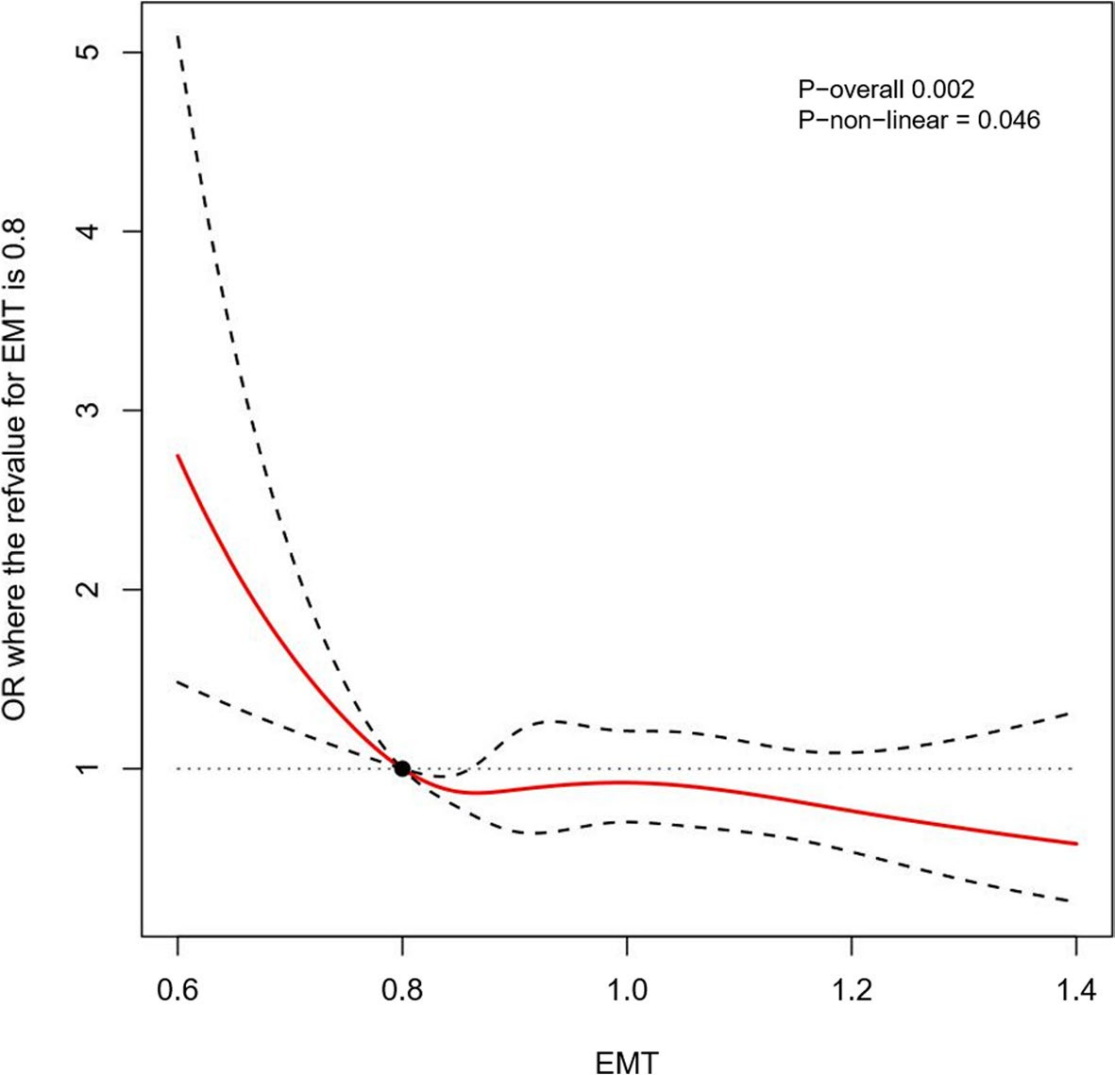
The restricted cubic splines for EMT in association with HDP rate in artificial cycle incorporating multivariate stepwise regression analysis model. (EMT = 0.8 cm as reference, knots = 5)

After subgroup stepwise logistic regression analysis based on endometrial preparation regimens (Table 3), the incidence of HDP increased significantly (aOR = 1.71; 95% CI, 1.22–2.33, *P* = 0.001) in patients with thin endometria prepared by an artificial cycle for FET, but conversely, patients in the natural cycle group with thick endometria suffered more greatly from HDP than the reference group (aOR = 1.59; 95% CI, 1.11–2.21, *P* = 0.008). On the other hand, a thick EMT in artificial cycles did not increase the HDP risk and the odds ratio was not significant for HDP incidence in natural cycles with thin EMT after multivariate logistic regression analysis (aOR = 1.02; 95% CI, 0.54–1.78, *P* = 0.940; aOR = 1.66; 95% CI, 0.94–2.74, *P* = 0.061, respectively). These results were remeasured by the restricted cubic splines of natural cycles and artificial cycles separately (Figs. 3 and 4).

**Table 3:** Subgroup multivariate logistic regression analysis of EMT based on endometrial preparation regimen (Stepwise logistic regression)

Variable	Artificial cycle		Natural cycle	
	OR (95% CI)	*P*-value	OR (95% CI)	*P*-value
Maternal age	1.01 (0.90–1.13)	0.843	1.23 (1.08–1.40)	0.002
Maternal BMI	1.23 (1.11–1.36)	< 0.001	1.39 (1.25–1.54)	< 0.001
Childbirth experience	0.64 (0.48–0.85)	0.002	0.46 (0.34–0.62)	< 0.001
**EMT**				
0.8 cm ≤ EMT ≤ 1.2 cm	Ref		Ref	
EMT < 0.8 cm	1.71 (1.22–2.33)	0.001	1.66 (0.94–2.74)	0.061
EMT > 1.2 cm	1.02 (0.54–1.78)	0.940	1.59 (1.11–2.21)	0.008
MAP	1.55 (1.38–1.74)	< 0.001	1.90 (1.66–2.18)	< 0.001
GDM	1.74 (1.25–2.38)	< 0.001	1.86 (1.28–2.65)	< 0.001
**Number of embryos**				
1	Ref		Ref	
> 1	0.72 (0.45–1.10)	0.151	0.81 (0.48–1.27)	0.382

*EMT* Endometrial thickness, *MAP* Mean arterial pressure, *GDM* Gestational diabetes mellitus

**Table 4:** The multivariate stepwise regression analysis model incorporating restricted cubic splines for EMT in association with HDP rate

EMT (cm)	Overall		Natural cycle		Artificial cycle	
	Unadjusted	Adjusted	Unadjusted	Adjusted	Unadjusted	Adjusted
0.6	2.42 (1.55–3.77)	2.58 (1.63–4.09)	1.69 (0.79–3.59)	1.71 (0.79–3.74)	2.38 (1.31–4.36)	2.75 (1.48–5.09)
0.7	1.93 (1.39–2.69)	2.03 (1.44–2.86)	1.48 (0.84–2.61)	1.50 (0.84–2.68)	1.89 (1.22–2.94)	2.10 (1.34–3.29)
0.8	Ref	Ref	Ref	Ref	Ref	Ref
0.9	0.86 (0.80–0.92)	0.86 (0.80–0.93)	0.88 (0.74–1.05)	0.88 (0.74–1.06)	0.89 (0.80–1.00)	0.87 (0.77–0.98)
1.0	0.85 (0.70–1.02)	0.90 (0.75–1.09)	0.89 (0.67–1.18)	0.88 (0.66–1.17)	1.00 (0.73–1.36)	0.91 (0.67–1.25)
1.1	0.78 (0.64–0.96)	0.89 (0.72–1.11)	1.01 (0.72–1.42)	0.96 (0.68–1.36)	1.05 (0.79–1.38)	0.90 (0.68–1.20)
1.2	0.72 (0.57–0.91)	0.86 (0.68–1.10)	1.09 (0.78–1.53)	1.00 (0.71–1.41)	0.97 (0.72–1.30)	0.82 (0.60–1.10)
1.3	0.81 (0.65–1.01)	1.02 (0.81–1.29)	1.31 (0.95–1.81)	1.21 (0.87–1.68)	0.85 (0.54–1.33)	0.71 (0.45–1.12)
1.4	0.95 (0.70–1.28)	1.24 (0.90–1.71)	1.62 (1.11–2.37)	1.54 (1.04–2.27)	0.75 (0.37–1.49)	0.62 (0.31–1.24)
1.5	1.10 (0.70–1.73)	1.51 (0.95–2.42)	2.01 (1.19–3.40)	1.96 (1.15–3.36)	–	–
1.6	1.28 (0.70–2.37)	1.84 (0.98–3.47)	2.50 (1.23–5.05)	2.50 (1.21–5.16)	–	–

*EMT* Endometrial thickness

## Discussion

HDP has received widespread attention owing to its negative impact on maternal and perinatal mortality. Hence, a retrospective cohort study of 13,458 single deliveries was performed to evaluate the association between EMT and HDP rate in FET cycles. We noted that both thin endometria and excessively thick endometria were identified as independent risk factors associated with HDP after adjusting for confounders. The non-linear dose-response relationship between EMT and HDP incidence in FET cycles was examined by restricted cubic spline.

Numerous studies have confirmed the risk of HDP with FET [10, 27], especially with programmed FET cycles, and the underlying mechanism is still unclear [19]. The main hypothesis is the absence of CL in artificial cycles that are commonly used for FET and this has been confirmed by both clinical and biological studies [28, 29]. The explanation is that CL produce not only estrogen and progesterone, but also vasoactive products including relaxin, vascular endothelial growth factor, and angiogenic metabolites of estrogen [30]. Therefore, programmed FET cycles are associated with a deficiency of these vasoactive products compared with FET cycles with CLs, including natural, modified natural, and stimulated cycles [29–31].

In addition to the absence of a CL, the clear etiology and pathophysiology of HDP is yet to be elucidated. It is now well known that placentation in humans is associated with unique vascular remodeling [32, 33]. Remodeling of the spiral arteries by extravillous trophoblasts (EVTs) is critical for adapting blood flow and nutrient transport to the developing fetus [34]. In pre-eclampsia, a major defect occurs in myometrial spiral artery remodeling [35–37]. The present literature suggests that the decidual environment critically modulates EVT function leading to reproductive success [38]. Does a thin or a thick EMT influence EVT processes and induce superficial implantation? More research is needed to elucidate the correlation between EMT and EVT invasion, implantation, and placentation. In particular, a thin EMT might result from uterine curettage or infection, which could induce microcirculation and local immune changes, as well as the inflammation. Recent studies have raised that these microenvironment changes would influence epigenetics progress in the placental formation by miRNAs expression [39–41]. Moreover, further large prospective studies on molecular biomarkers in excessive thin or deep endometrium, like soluble fms-like tyrosine kinase-1 (sFlt-1), placental grow factor (PlGF), natural killer (NK) cells, would help to clarify the pathophysiology of HDP [42–45].

The EMT is a priority in FET cycles and is relatively controllable. In the present study, the average EMT in artificial cycles was significantly thinner than in natural cycles. In an artificial cycle, once the EMT reached the basic criteria (around 0.8 cm), luteal support would usually be given rather than extending the proliferation phase to achieve a better thickness. However, in a natural cycle, the endometrium usually reaches an optimum thickness coincident with dominant follicle development and rupture. This might explain, why the endometrium was thicker in natural cycles than artificial cycles in clinical practice. The present study offers important evidence for positively adjusting EMT in FET cycles to improve perinatal outcome, especially in artificial cycles.

It is interesting that the influence of EMT on the incidence of HDP appeared in different patterns according to the two endometrial preparation regimens. The hypothesis is that the presence of CL might differently interact with endometria of varying thickness during decidualization, implantation, and EVT invasion. The etiology and physiology of this phenomenon urgently needs to be revealed. Nevertheless, the relatively small sample size of patients with a thick EMT in the artificial cycle group may have contributed to the result, to some extent. As shown in Table 1, in the thick EMT group, only 14.31% of patients were prepared by the artificial method.

Clinicians need to pay attention to those patients undergoing FET using an artificial method who have a thin EMT. Alternately, a natural cycle might be a better choice over an artificial protocol for patients with an inherently thin EMT. Furthermore, subgroup analysis results from the natural cycle group also raises questions as to whether women having a spontaneous pregnancy are influenced by EMT in the same way.

Another interesting question is whether differences in obstetrics and perinatal outcomes between fresh transfer and FET cycles, including HDP incidence, are partly induced by the difference in EMT. EMT might reach a relatively thicker state in controlled ovarian stimulation because of the extraordinary higher estradiol levels. Future studies are needed to illustrate this point.

To the best of our knowledge, this study is the first to identify the non-linear dose-response relationship between EMT and HDP risk in FET cycles. Unfortunately, in most published literature, EMT variability is commonly missing in analyses of association between the risk of HDP and FET treatment parameters. While in some other studies, EMT was taken as the covariates without stratification and failed to be estimated as a significant risk factor. Our study had three advantages compared to other studies. First, the large sample size of this single-center study, in which clinical and IVF laboratory practices were uniformly controlled, reduced the potential bias and enhanced the statistical power. Second, we had strict inclusion and exclusion criteria. Finally, we adjusted a variety of potential predictors to minimize the potential effects of confounding factors. There were several limitations in our study. For the retrospective analysis, there were some intrinsic defects that could not be avoided. It is also important to note that some patients with a thin or thick endometrium proceeded with ET, while other patients canceled transfer. This might have created some bias and therefore may not reflected the true effect of thin or thick endometria. In addition, confounding factors known to influence pregnancy outcome, such as smoking and HDP history in previous pregnancies, were not recorded in the database.

## Conclusion

The results of this study confirm that EMT in FET cycles is an independent risk factor on the development of pregnancy-induced hypertension. Clinicians should pay more attention to EMT during FET cycles for the benefit of maternal and child health. More research is needed to clarify the relationship between EMT and EVT invasion.

## Data Availability

The datasets used and/or analyzed during the current study are available from the corresponding author on reasonable request.

## Abbreviations

HDP: Hypertensive disorders of pregnancy
EMT: Endometrial thickness
FET: Frozen-thawed embryo transfer
ART: Assisted reproductive technology
ET: Fresh embryo transfer
IVF/ICSI: In vitro fertilization/intracytoplasmic sperm injection
LGA: Large for gestational age
CL: Corpus luteum
ANOVA: One-way analysis of variance
OR: Odds ratio
95%CI: 95% confidence intervals
aOR: Adjusted odds ratio
BMI: Body mass index
PCOS: Polycystic ovary syndrome
MAP: Mean arterial pressure
GDM: Gestational diabetes mellitus
OGTT: Oral glucose tolerance test
ROC curve: Receiver operating characteristic curve
AUC: Area under the curve
sFlt-1: Soluble fms-like tyrosine kinase-1
PlGF: Placental grow factor
NK cell: Natural killer cell

## References

[1] Chen M, Heilbronn LK (2017). The health outcomes of human offspring conceived by assisted reproductive technologies (ART). J Dev Orig Health Dis.

[2] Henningsen AKA, Pinborg A, Lidegaard Ø, Vestergaard C, Forman JL, Andersen AN (2011). Perinatal outcome of singleton siblings born after assisted reproductive technology and spontaneous conception: Danish national sibling-cohort study. Fertil Steril.

[3] Sha T, Yin X, Cheng W, Massey IY (2018). Pregnancy-related complications and perinatal outcomes resulting from transfer of cryopreserved versus fresh embryos in vitro fertilization: a meta-analysis. Fertil Steril.

[4] Pelkonen S, Koivunen R, Gissler M, Nuojua-Huttunen S, Suikkari AM, Hydén-Granskog C, Martikainen H, Tiitinen A, Hartikainen AL (2010). Perinatal outcome of children born after frozen and fresh embryo transfer: The Finnish cohort study.

[5] Shavit M, Miller N, Schreiber H, Asali A, Ravid D, Harlev A, Levitas E, Har-Vardi I, Berkovitz A (2019). Twin pregnancies and perinatal outcomes: a comparison between fresh and frozen embryo transfer: a two-centre study. Reprod Biomed Online.

[6] Glujovsky D, Pesce R, Sueldo C, Quinteiro Retamar AM, Hart RJ, Ciapponi A (2020). Endometrial preparation for women undergoing embryo transfer with frozen embryos or embryos derived from donor oocytes.

[7] Jing S, Luo K, He H, Lu C, Zhang S, Tan Y, Gong F, Lu G, Lin G (2016). Obstetric and neonatal outcomes in blastocyst-stage biopsy with frozen embryo transfer and cleavage-stage biopsy with fresh embryo transfer after preimplantation genetic diagnosis/screening. Fertil Steril.

[8] Bhattacharya S (2016). Maternal and perinatal outcomes after fresh versus frozen embryo transfer—what is the risk-benefit ratio?. Fertil Steril.

[9] Acharya KS, Acharya CR, Bishop K, Harris B, Raburn D, Muasher SJ (2018). Freezing of all embryos in in vitro fertilization is beneficial in high responders, but not intermediate and low responders: an analysis of 82,935 cycles from the Society for Assisted Reproductive Technology registry. Fertil Steril.

[10] Chen Z-J, Shi Y, Sun Y, Zhang B, Liang X, Cao Y, Yang J, Liu J, Wei D, Weng N (2016). Fresh versus frozen embryos for infertility in the polycystic ovary syndrome. N Engl J Med.

[11] Maheshwari A, Pandey S, Raja EA, Shetty A, Hamilton M, Bhattacharya S (2018). Is frozen embryo transfer better for mothers and babies? Can cumulative meta-analysis provide a definitive answer?. Hum Reprod Update.

[12] Imudia AN, Awonuga AO, Doyle JO, Kaimal AJ, Wright DL, Toth TL, Styer AK (2012). Peak serum estradiol level during controlled ovarian hyperstimulation is associated with increased risk of small for gestational age and preeclampsia in singleton pregnancies after in vitro fertilization. Fertil Steril.

[13] Royster GD, Krishnamoorthy K, Csokmay JM, Yauger BJ, Chason RJ, DeCherney AH, Wolff EF, Hill MJ (2016). Are intracytoplasmic sperm injection and high serum estradiol compounding risk factors for adverse obstetric outcomes in assisted reproductive technology?. Fertil Steril.

[14] Von Versen-Hoÿnck F, Narasimhan P, Selamet Tierney ES, Martinez N, Conrad KP, Baker VL, Winn VD (2019). Absent or excessive corpus luteum number is associated with altered maternal vascular health in early pregnancy. Hypertension.

[15] Liu KE, Hartman M, Hartman A, Luo ZC, Mahutte N (2018). The impact of a thin endometrial lining on fresh and frozen-thaw IVF outcomes: an analysis of over 40 000 embryo transfers. Hum Reprod.

[16] Casper RF (2020). Frozen embryo transfer: evidence-based markers for successful endometrial preparation. Fertil Steril.

[17] Guo Z, Xu X, Zhang L, Zhang L, Yan L, Ma J (2020). Endometrial thickness is associated with incidence of small-for-gestational-age infants in fresh in vitro fertilization–intracytoplasmic sperm injection and embryo transfer cycles. Fertil Steril.

[18] Liu X, Wang J, Fu X, Li J, Zhang M, Yan J, Gao S, Ma J (2021). Thin endometrium is associated with the risk of hypertensive disorders of pregnancy in fresh IVF/ICSI embryo transfer cycles: a retrospective cohort study of 9,266 singleton births. Reprod Biol Endocrinol.

[19] Wang Z, Liu H, Song H, Li X, Jiang J, Sheng Y, Shi Y (2020). Increased risk of pre-eclampsia after frozen-thawed embryo transfer in programming cycles.

[20] Yx L, Tz S, Mq L, Zhou L, Ge P, Hn L, Dx Z (2020). Is vanishing twin syndrome associated with adverse obstetric outcomes of ART singletons? A systematic review and meta-analysis. J Assist Reprod Genet.

[21] Hypertension G (2020). Gestational Hypertension and Preeclampsia: ACOG Practice Bulletin, Number 222.

[22] Azziz R, Tarlatzis R, Dunaif A, Ibanez L, Pugeat M, Taylor A, Fauser CJM, Medicine R (2004). Revised 2003 consensus on diagnostic criteria and long-term health risks related to polycystic ovary syndrome. Fertil Steril.

[23] Escobar F (2018). Polycystic ovary syndrome: definition, aetiology, diagnosis and treatment.

[24] Wehrwein EA, Joyner MJ (2013). Regulation of blood pressure by the arterial baroreflex and autonomic nervous system. Handb Clin Neurol.

[25] Mayrink J, Souza RT, Feitosa FE, Rocha Filho EA, Leite DF, Vettorazzi J, Calderon IM, Costa ML, Kenny L, Baker P (2019). Mean arterial blood pressure: Potential predictive tool for preeclampsia in a cohort of healthy nulliparous pregnant women.

[26] Metzger BE (2010). International Association of Diabetes and Pregnancy Study Groups recommendations on the diagnosis and classification of hyperglycemia in pregnancy. Diabetes Care.

[27] Wei D, Liu JY, Sun Y, Shi Y, Zhang B, Liu JQ, Tan J, Liang X, Cao Y, Wang Z (2019). Frozen versus fresh single blastocyst transfer in ovulatory women: a multicentre, randomised controlled trial. Lancet.

[28] Von Versen-Hoÿnck F, Schaub AM, Chi YY, Chiu KH, Liu J, Lingis M, Stan Williams R, Rhoton-Vlasak A, Nichols WW, Fleischmann RR (2019). Increased preeclampsia risk and reduced aortic compliance with in vitro fertilization cycles in the absence of a corpus luteum. Hypertension.

[29] Singh B, Reschke L, Segars J, Baker VL (2020). Frozen-thawed embryo transfer: the potential importance of the corpus luteum in preventing obstetrical complications. Fertil Steril.

[30] Conrad KP, Baker VL (2013). Corpus luteal contribution to maternal pregnancy physiology and outcomes in assisted reproductive technologies.

[31] von Versen-Höynck F, Strauch NK, Liu J, Chi YY, Keller-Woods M, Conrad KP, Baker VL (2019). Effect of mode of conception on maternal serum Relaxin, creatinine, and sodium concentrations in an infertile population. Reprod Sci.

[32] Pijnenborg R, Vercruysse L, Hanssens M (2006). The uterine spiral arteries in human pregnancy: facts and controversies. Placenta.

[33] Burton GJ, Woods AW, Jauniaux E, Kingdom JCP (2009). Rheological and physiological consequences of conversion of the maternal spiral arteries for uteroplacental blood flow during human pregnancy. Placenta.

[34] Sato Y (2020). Endovascular trophoblast and spiral artery remodeling. Mol Cell Endocrinol.

[35] Lyall F, Robson SC, Bulmer JN (2013). Spiral artery remodeling and trophoblast invasion in preeclampsia and fetal growth restriction relationship to clinical outcome. Hypertension.

[36] Conrad KP (2011). Pregnancy: implications for preeclampsia.

[37] Phipps EA, Thadhani R, Benzing T, Karumanchi SA (2019). Pre-eclampsia: pathogenesis, novel diagnostics and therapies. Nat Rev Nephrol.

[38] Pollheimer J, Vondra S, Baltayeva J, Beristain AG, Knöfler M (2018). Regulation of placental extravillous trophoblasts by the maternal uterine environment. Front Immunol.

[39] Chiofalo B, Lagana AS, Vaiarelli A, La Rosa VL, Rossetti D, Palmara V, Valenti G, Rapisarda AMC, Granese R, Sapia F (2017). Do miRNAs play a role in fetal growth restriction? A fresh look to a busy corner. Biomed Res Int.

[40] Lagana AS, Vitale SG, Sapia F, Valenti G, Corrado F, Padula F, Rapisarda AMC, D'Anna R (2018). miRNA expression for early diagnosis of preeclampsia onset: hope or hype?. J Matern Fetal Neonatal Med.

[41] Cirkovic A, Stanisavljevic D, Milin-Lazovic J, Rajovic N, Pavlovic V, Milicevic O, Savic M, Kostic Peric J, Aleksic N, Milic N (2021). Preeclamptic women have disrupted placental microRNA expression at the time of preeclampsia diagnosis: meta-analysis. Front Bioeng Biotechnol.

[42] Richard J, Levine MD, Maynard SE, Qian C, Lim K-H, England LJ, Yu KF, Schisterman EF, Thadhani R, Sachs BP, Epstein FH, Sibai BM, Sukhatme VP, Karumanchi SA (2004). Circulating angiogenic factors and the risk of preeclampsia. N Engl J Med.

[43] Richard J, Levine MD, Lam C, Qian C, Yu KF, Maynard SE, Sachs BP, Sibai BM, Epstein FH, Romero R, Thadhani R, Karumanchi SA (2006). Soluble Endoglin and Other Circulating Antiangiogenic Factors in Preeclampsia. N Engl J Med.

[44] Lagana AS, Favilli A, Triolo O, Granese R, Gerli S (2016). Early serum markers of pre-eclampsia: are we stepping forward?. J Matern Fetal Neonatal Med.

[45] Lagana AS, Giordano D, Loddo S, Zoccali G, Vitale SG, Santamaria A, Buemi M, D'Anna R (2017). Decreased endothelial progenitor cells (EPCs) and increased natural killer (NK) cells in peripheral blood as possible early markers of preeclampsia: a case-control analysis. Arch Gynecol Obstet.

